# Crystal structure, computational study and Hirshfeld surface analysis of ethyl (2*S*,3*R*)-3-(3-amino-1*H*-1,2,4-triazol-1-yl)-2-hy­droxy-3-phenyl­propano­ate

**DOI:** 10.1107/S2056989019015743

**Published:** 2019-11-26

**Authors:** Abdelkader Ben Ali, Youness El Bakri, Chin-Hung Lai, Jihad Sebhaoui, Lhoussaine El Ghayati, El Mokhtar Essassi, Joel T. Mague

**Affiliations:** a Laboratoire de Chimie Appliquée des Matériaux, Centres des Sciences des Matériaux, Faculty of Sciences, Mohammed V University in Rabat, Avenue Ibn, Battouta, BP 1014, Rabat, Morocco; b Laboratoire de Chimie Organique Hétérocyclique, Centre de Recherche des Sciences des Médicaments, URAC 21, Pôle de Compétence Pharmacochimie, Av Ibn Battouta, BP 1014, Faculté des Sciences, Université Mohammed V, Rabat, Morocco; c Department of Medical Applied Chemistry, Chung Shan Medical University, Taichung 40241, Taiwan; d Department of Medical Education, Chung Shan Medical University Hospital, Taichung 40241, Taiwan; e Department of Chemistry, Tulane University, New Orleans, LA 70118, USA

**Keywords:** crystal structure, triazole, hydrogen bond, C—H⋯π(ring) inter­action, Hirshfeld surface analysis, computational chemistry

## Abstract

In the title mol­ecule, C_13_H_16_N_4_O_3_, the mean planes of the phenyl and triazole rings are nearly perpendicular to one another as a result of the intra­molecular C—H⋯O and C—H⋯π(ring) inter­actions. In the crystal, layers parallel to (101) are generated by O—H⋯N, N—H⋯O and N—H⋯N hydrogen bonds. The layers are connected by inversion-related pairs of C—H⋯O hydrogen bonds. The experimental mol­ecular structure is close to the gas-phase geometry-optimized structure calculated by DFT methods. Hirshfeld surface analysis indicates that the most important inter­action involving hydrogen in the title compound is the H⋯H contact. The contribution of the H⋯O, H⋯N, and H⋯H contacts are 13.6, 16.1, and 54.6%, respectively.

## Abstract 2

The mean planes of the phenyl and triazole rings are nearly perpendicular to one another as a result of the intra­molecular C—H⋯O and C—H⋯π(ring) inter­actions. In the crystal, layers parallel to (101) are generated by O—H⋯N, N—H⋯O and N—H⋯N hydrogen bonds. The layers are connected by inversion-related pairs of C—H⋯O hydrogen bonds.

## Chemical context   

The triazole ring system has attracted considerable inter­est among synthetic organic chemists and those dealing with medicinal compounds because of its versatile potential to inter­act with biological systems (Martins *et al.*, 2015 ▸). Many of its derivatives are important as agrochemicals (Dayan *et al.*, 2000 ▸; Huang *et al.*, 2006 ▸; Ling *et al.*, 2007 ▸). There is also a continuing need for the development of new drugs as those currently available are becoming ineffective because of the drug resistance developed by pathogens. Moreover, life-threatening infections caused by pathogenic fungi are becoming increasingly very common (Leather & Wingard, 2006 ▸; Walsh *et al.*, 2004 ▸; Chai *et al.*, 2011 ▸). Triazole compounds have shown great efficacy against fungal infections. In 1944, Woolly discovered the excellent anti­fungal properties of azole derivatives, which led to the development of fluconazole, variconazole, albaconazole and itraconazole (Dismukes *et al.*, 2000 ▸; Zonios *et al.*, 2008 ▸; Gupta *et al.*, 2003 ▸). Further structural modifications of this ring system are expected to result in potential candidates for anti­fungal agents. These modifications use different functionalities such as aliphatic chains, aromatic rings, heterocyclic ring systems *etc.* (Calderone *et al.*, 2008 ▸; Kim *et al.*, 2010 ▸; Giffin *et al.*, 2008 ▸; Wang *et al.*, 2005 ▸). As a continuation of our research on the synthesis, functionalization, physico-chemical and biological properties of triazole derivatives (El Bakri *et al.*, 2018 ▸, 2019*a* ▸,*b* ▸,*c* ▸), we report herein on the crystal structure, DFT calculations and Hirshfeld surface analysis of ethyl (2*S*,3*R*)-3-(3-amino-1*H*-1,2,4-triazol-1-yl)-2-hy­droxy-3-phenyl­propano­ate (**1**).
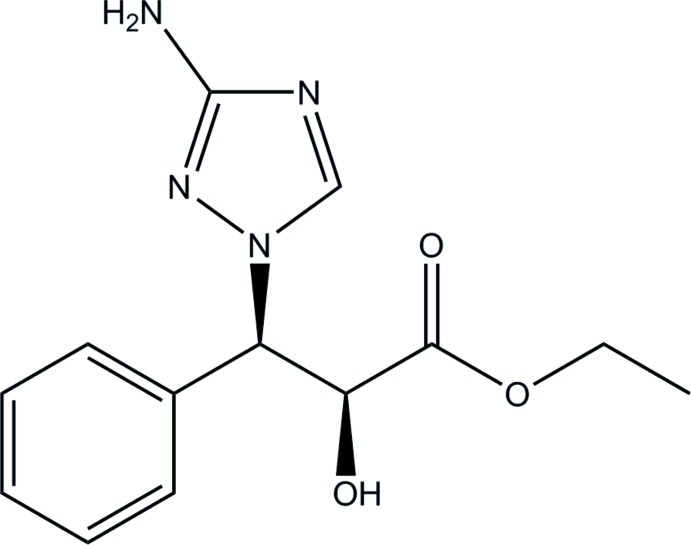


## Structural commentary   

**Figure 1:**
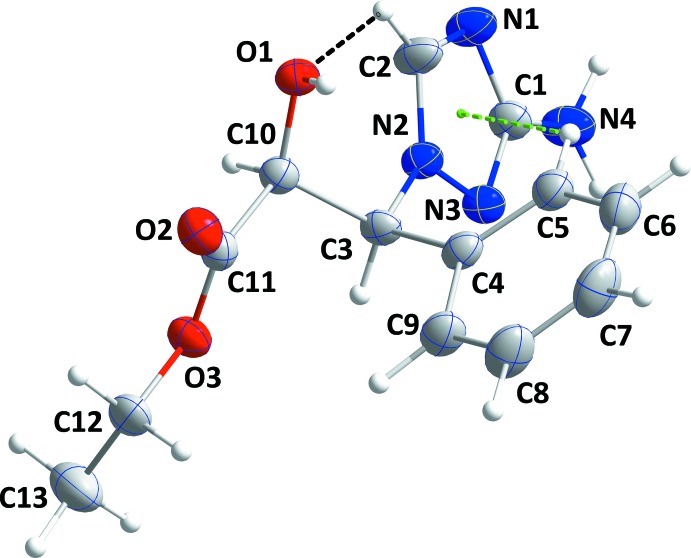
The title mol­ecule with the labelling scheme and 50% probability displacement ellipsoids. The intra­molecular C—H⋯O hydrogen bond is shown by a black dashed line while the C—H⋯π(ring) inter­action is shown by a green dashed line.

The conformation of the mol­ecule is controlled in part by two intra­molecular inter­actions, a C2—H2⋯O1 hydrogen bond and a C—H⋯π(ring) inter­action between C5—H5 and the triazole ring (Table 1 ▸ and Fig. 1 ▸). This leads to a dihedral angle of 87.12 (4)° between the phenyl and triazole rings. Atoms N4 and C3 are displaced from the mean plane of the triazole ring by 0.046 (1) and −0.056 (1) Å, respectively. All bond distances and inter­bond angles are as expected for the formulation given.

## Supra­molecular features   

**Figure 2:**
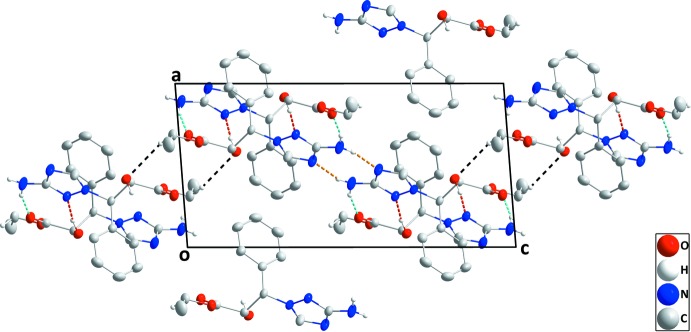
The packing viewed along the *b*-axis direction. O—H⋯N, N—H⋯O, N—H⋯N and C—H⋯O hydrogen bonds are shown, respectively, by red, light-blue, orange and black dashed lines.

In the crystal, O1—H1⋯N3, N4—H4*A*⋯O2 and N4—H4*B*⋯N1 hydrogen bonds (Table 1 ▸) form layers of mol­ecules parallel to (101) (see Fig. 2 ▸), which are joined by inversion-related pairs of C12—H12*B*⋯O1 hydrogen bonds (Table 1 ▸ and Fig. 2 ▸).

## Database survey   

Searches of the CSD (Version 5.40, updated to September 2019; Groom *et al.*, 2016 ▸) with two different search fragments were performed. The first, with 3-amino-1*H*-1,2,4-triazole as the search fragment, found three structures in which a side chain is bound to the nitro­gen atom in the 1-position of the triazole ring (N2 in **1**), namely 4-(3-amino-1*H*-1,2,4-triazol-1-yl)-4-methyl­pentan-2-one (QISROC; Zemlyanaya *et al.*, 2018 ▸), 1-(3-amino-1*H*-1,2,4-triazol-1-yl)-3,3-di­methyl­butan-2-one (VATPEO; Cai *et al.*, 2017 ▸) and 3-amino-1-guanyl-1,2,4-triazole dinitramide (YOPDAJ; Zeng *et al.*, 2008 ▸). The triazole ring in each of these is essentially planar and the distances of the corresponding C and N substituent atoms from the mean plane of the triazole ring are comparable to those observed for **1**.

The second search, using 1-benzyl-1*H*-1,2,4-triazole as the search fragment, found fifteen structures, but in most of these the phenyl group is oriented with the line joining the *ortho* carbon atoms approximately parallel to that joining the atoms in the triazole ring corresponding to C2 and N3 in Fig. 1 ▸, so that there is an intra­molecular C—H⋯π(ring) inter­action is not possible. Those in which this inter­action is possible are (+)-6-[(4-chloro­phen­yl)(1*H*-1,2,4-triazol-1-yl)meth­yl]-1-methyl-1*H*-benzotriazole (HALHOR; Peeters *et al.*, 1993 ▸), (+)-6-[(4-chloro­phen­yl)(4-azonia-1*H*-1,2-diazol-1-yl)meth­yl]-1-methyl-1*H*-benzotriazole bromide monohydrate (HALHUX; Peeters *et al.*, 1993 ▸), 5,6-bis­{4-methyl-2,6-bis­[(1*H*-1,2,4-triazol-1-yl)meth­yl]phen­oxy}pyrazine-2,3-dicarbo­nitrile monohydrate (NEJFIU; Ghazal *et al.*, 2017 ▸) and 4,4′-(1*H*-1,2,4-triazol-1-yl)methyl­enebis(benzo­nitrile) (UKAKIA; Xu *et al.*, 2002 ▸). The H⋯centroid distances and C—H⋯centroid angles for these are: HALHOR: 2.94 Å, 111°; HALHUX: 2.78 Å, 124°; NEJFIU: 2.92 Å, 153° and 2.66 Å, 127°; UKAKIA: 2.83 Å, 126°. The geometries of all of the C—H⋯π(ring) inter­actions in these mol­ecules, except for the first of the two inter­actions listed for NEJFIU, are comparable to that found in **1**.

## Theoretical studies   

### calculation of the electronic structure   

The structure in the gas phase of **1** was optimized by means of density functional theory. The DFT calculation was performed by the hybrid B3LYP method, which is based on the idea of Becke and considers a mixture of the exact (HF) and DFT exchange utilizing the B3 functional, together with the LYP correlation functional (Becke, 1993 ▸; Lee *et al.*, 1988 ▸; Miehlich *et al.*, 1989 ▸). In conjunction with the basis set def2-SVP, the B3LYP calculation was performed (Weigend & Ahlrichs, 2005 ▸). After obtaining the converged geometry, the harmonic vibrational frequencies were calculated at the same theoretical level to confirm the number of imaginary frequencies is zero for the stationary point. Both the geometry optimization and harmonic vibrational frequency analysis of **1** were performed using the *Gaussian 16* program (Frisch *et al.*, 2016 ▸).

### comparison between the gas- and solid-phase geometries   

**Figure 3:**
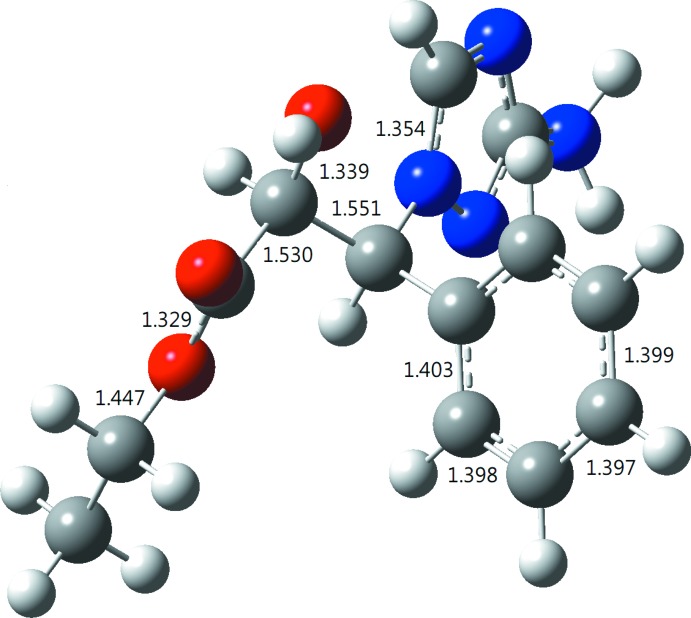
The B3LYP-optimized geometry (Å) of the title compound.

From a comparison of selected geometrical parameters obtained from the B3LYP geometry optimization for **1** (Fig. 3 ▸) with those from the crystallographic study (Table 2 ▸), it is evident that the B3LYP-optimized geometry shows little deviation from the X-ray structure. To qu­antify the difference between the calculated and experimental geometries, the structure comparer built into the *ChemCraft* software (https://www.chemcraftprog.com) was used to obtain their r.m.s. deviation. A weighted r.m.s.d. of 0.5684 was obtained with r.m.s. deviations of 0.7365, 0.4474, 0.1926, and 0.2606 for the H, C, N and O atoms, respectively.

### Hirshfeld surface analysis   

Both the definition of a mol­ecule in a condensed phase and the recognition of distinct entities in mol­ecular liquids and crystals are fundamental concepts in chemistry. Based on Hirshfeld’s partitioning scheme, Spackman *et al.* (1997 ▸) proposed a method to divide the electron distribution in a crystalline phase into mol­ecular fragments (Spackman & Byrom, 1997 ▸; McKinnon *et al.*, 2004 ▸; Spackman & Jayatilaka, 2009 ▸). Their proposed method partitioned the crystal into regions where the electron distribution of a sum of spherical atoms for the mol­ecule dominates over the corresponding sum of the crystal. As it is derived from Hirshfeld’s stockholder partitioning, the mol­ecular surface is named as the Hirshfeld surface. In this study, the Hirshfeld surface analysis of **1** was performed using *CrystalExplorer* (Turner *et al.*, 2017 ▸).

**Figure 4:**
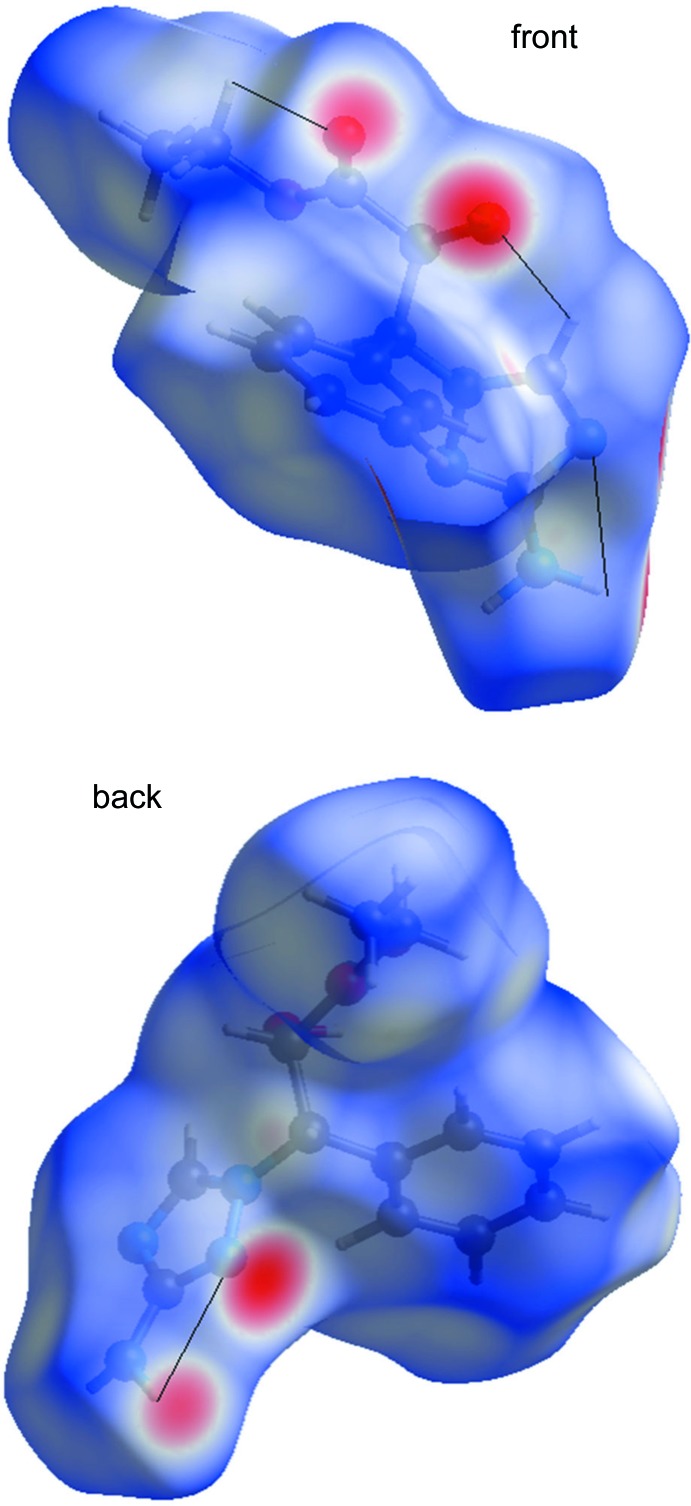
The *d*_norm_ Hirshfeld surface of the title compound (red: negative, white: zero, blue: positive; scale: −0.6530 to 1.3260 a.u.).

**Figure 5:**
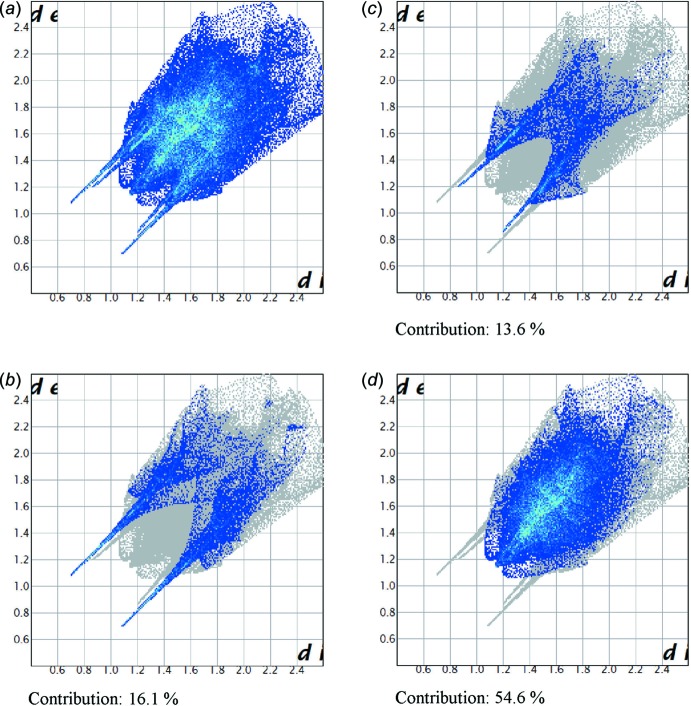
The two-dimensional fingerprint plots of the title compound (*a*) all, and delineated into (*b*) H⋯N, (*c*) H⋯O and (*d*) H⋯H contacts.

The standard resolution mol­ecular Hirshfeld surface (*d*_norm_) of **1** is depicted in Fig. 4 ▸. This surface can be used to identify very close inter­molecular inter­actions. The value of *d*_norm_ is negative (positive) when inter­molecular contacts are shorter (longer) than the van der Waals radii. The red regions on the surface represent closer contacts with a negative *d*_norm_ value while the blue regions represent longer contacts with a positive *d*_norm_ value while, the white regions represent contacts equal to the van der Waals separation and have a *d*_norm_ value of zero. As depicted in Fig. 4 ▸, the important inter­actions in **1** are H⋯O and H⋯N hydrogen bonds. In order to understand the relative importance of H⋯O hydrogen bonds *versus* H⋯N hydrogen bonds, we calculated the two-dimensional fingerprint plots for **1** (Fig. 5 ▸), which highlight particular atom-pair contacts and enable the separation of contributions from different inter­action types that overlap in the full fingerprint. The most important inter­action involving hydrogen in **1** is the H⋯H contact. The contributions of the H⋯O, H⋯N, and H⋯H contact are 13.6%, 16.1% and 54.6%, respectively.

## Synthesis and crystallization   

A mixture of 3-amino-1,2,4-triazole (2 g, 23.8 mmol) and ethyl 3-phenyl­glycidate (4.5 mL, 32.8 mmol) in *n-*butanol (20 mL) was refluxed for 24 h. After completion of the reaction (TLC indicated complete consumption of reactants), the solvents were removed *in vacuo*. The purified product was recrystallized from ethanol solution to afford **1** as colourless crystals. ^1^H NMR (300 MHz, DMSO-*d*_6_), δ(ppm): 1.77 (*s*, 3H, CH_3_),7.66 (*q*, 2H, CH_2_), 5.21 (*d*, 1H, CH), 5.82 (*d*, 1H, CH), 6.20 (*s*, 1H, OH), 6.62 (*s*, 2H, NH_2_), 7.28–7.32 (CH_Ar_), 8.32 (*s*, 1H, CH_triazolic_).^13^C NMR (75 MHz, DMSO-*d*_6_) δ (ppm): δ 15.6, 63.8, 70.3, 82.02, 129.2, 130.6, 131.1, 145.9, 146.8, 164.5, 172.9. HRMS (EI). Calculated for C_13_H_16_N_4_O_3_: [*M* + H^+^] = 277.12. Found: [*M* + H^+^] = 277.30. Elemental analysis: calculated: C, 56.51%; H, 5.84%; N, 20.28%; O, 17.37%, found: C, 56.76%; H, 4.16%; N, 19.94%; O, 19.14%.

## Refinement   

Crystal data, data collection and structure refinement details are summarized in Table 3 ▸.

## Supplementary Material

Crystal structure: contains datablock(s) global, I. DOI: 10.1107/S2056989019015743/tx2015sup1.cif

Structure factors: contains datablock(s) I. DOI: 10.1107/S2056989019015743/tx2015Isup2.hkl

Supporting information file. DOI: 10.1107/S2056989019015743/tx2015Isup3.cdx

Supporting information file. DOI: 10.1107/S2056989019015743/tx2015Isup4.cml

CCDC reference: 1967185

Additional supporting information:  crystallographic information; 3D view; checkCIF report

## Figures and Tables

**Table 1:** Hydrogen-bond geometry (Å, °) *Cg*1 is the centroid of the triazole ring.

*D*—H⋯*A*	*D*—H	H⋯*A*	*D*⋯*A*	*D*—H⋯*A*
O1—H1⋯N3^i^	0.881 (18)	1.887 (18)	2.7417 (12)	162.9 (16)
N4—H4*A*⋯O2^ii^	0.887 (16)	2.182 (17)	3.0317 (14)	160.2 (13)
N4—H4*B*⋯N1^iii^	0.898 (18)	2.127 (18)	3.0066 (15)	166.1 (15)
C2—H2⋯O1	0.954 (16)	2.257 (16)	2.8665 (14)	120.9 (12)
C12—H12*B*⋯O1^iv^	1.000 (15)	2.569 (15)	3.4225 (15)	143.2 (11)
C5—H5⋯*Cg*1	0.982 (14)	2.856 (14)	3.4816 (13)	122.3 (10)

Symmetry codes: (i) 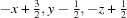; (ii) 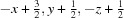; (iii) 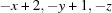; (iv) 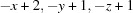.

**Table 2:** Bond lengths and angles (Å, °) in the B3LYP-optimized and the X-ray structures

	B3LYP	X-ray		B3LYP	X-ray
N1—C1	1.365	1.3648 (15)	O1—C10	1.399	1.3968 (13)
N1—C2	1.321	1.3242 (15)	O2—C11	1.210	1.2079 (14)
N2—C3	1.459	1.4609 (13)	O3—C11	1.329	1.3277 (14)
N2—C22	1.354	1.3319 (15)	O3—C12	1.447	1.4697 (13)
N2—N3	1.364	1.3794 (13)	C4—C9	1.403	1.3992 (16)
N3—C1	1.328	1.3288 (14)	C8—C7	1.397	1.380 (2)
N4—C1	1.377	1.3610 (15)	C8—C9	1.398	1.3867 (18)
C3—C4	1.523	1.5171 (15)	C10—C11	1.530	1.5278 (15)
C3—C10	1.551	1.5522 (15)			
					
C2—N2—N3	109.4	108.99 (9)	N1—C1—N3	114.9	114.34 (10)
O2—C11—O3	125.0	125.05 (10)	C10—C11—O3	113.4	111.04 (9)

**Table 3:** Experimental details

Crystal data	
Chemical formula	C_13_H_16_N_4_O_3_
*M*_r_	276.30
Crystal system, space group	Monoclinic, *P*2_1_/*n*
Temperature (K)	150
*a*, *b*, *c* (Å)	8.4766 (2), 9.4841 (2), 16.9904 (3)
β (°)	94.308 (1)
*V* (Å^3^)	1362.05 (5)
*Z*	4
Radiation type	Cu *K*α
μ (mm^−1^)	0.82
Crystal size (mm)	0.34 × 0.22 × 0.09

Data collection	
Diffractometer	Bruker D8 VENTURE PHOTON 100 CMOS
Absorption correction	Multi-scan (*SADABS*; Krause *et al.*, 2015 ▸)
*T*_min_, *T*_max_	0.83, 0.93
No. of measured, independent and observed [*I* > 2σ(*I*)] reflections	10203, 2718, 2485
*R*_int_	0.027
(sin θ/λ)_max_ (Å^−1^)	0.625

Refinement	
*R*[*F* ^2^ > 2σ(*F* ^2^)], *wR*(*F* ^2^), *S*	0.033, 0.084, 1.05
No. of reflections	2718
No. of parameters	246
H-atom treatment	All H-atom parameters refined
Δρ_max_, Δρ_min_ (e Å^−3^)	0.22, −0.16

Computer programs: *APEX3* and *SAINT* (Bruker, 2016 ▸), *SHELXT* (Sheldrick, 2015*a* ▸), *SHELXL2018* (Sheldrick, 2015*b* ▸), *DIAMOND* (Brandenburg & Putz, 2012 ▸) and *SHELXTL* (Sheldrick, 2008 ▸).

## supplementary crystallographic information

### Crystal data

**Table:**

C_13_H_16_N_4_O_3_	*F*(000) = 584
*M**_r_* = 276.30	*D*_x_ = 1.347 Mg m^−^^3^
Monoclinic, *P*2_1_/*n*	Cu *K*α radiation, λ = 1.54178 Å
*a* = 8.4766 (2) Å	Cell parameters from 8615 reflections
*b* = 9.4841 (2) Å	θ = 2.6–74.5°
*c* = 16.9904 (3) Å	µ = 0.82 mm^−^^1^
β = 94.308 (1)°	*T* = 150 K
*V* = 1362.05 (5) Å^3^	Thick plate, colourless
*Z* = 4	0.34 × 0.22 × 0.09 mm

### Data collection

**Table:**

Bruker D8 VENTURE PHOTON 100 CMOS diffractometer	2718 independent reflections
Radiation source: INCOATEC IµS micro–focus source	2485 reflections with *I* > 2σ(*I*)
Mirror monochromator	*R*_int_ = 0.027
Detector resolution: 10.4167 pixels mm^-1^	θ_max_ = 74.5°, θ_min_ = 5.2°
ω scans	*h* = −9→10
Absorption correction: multi-scan (*SADABS*; Krause *et al.*, 2015)	*k* = −11→11
*T*_min_ = 0.83, *T*_max_ = 0.93	*l* = −20→21
10203 measured reflections	

### Refinement

**Table:**

Refinement on *F*^2^	Secondary atom site location: difference Fourier map
Least-squares matrix: full	Hydrogen site location: difference Fourier map
*R*[*F*^2^ > 2σ(*F*^2^)] = 0.033	All H-atom parameters refined
*wR*(*F*^2^) = 0.084	*w* = 1/[σ^2^(*F*_o_^2^) + (0.0378*P*)^2^ + 0.3844*P*] where *P* = (*F*_o_^2^ + 2*F*_c_^2^)/3
*S* = 1.05	(Δ/σ)_max_ < 0.001
2718 reflections	Δρ_max_ = 0.22 e Å^−^^3^
246 parameters	Δρ_min_ = −0.16 e Å^−^^3^
0 restraints	Extinction correction: *SHELXL2018* (Sheldrick, 2015*b*), Fc^*^=kFc[1+0.001xFc^2^λ^3^/sin(2θ)]^-1/4^
Primary atom site location: dual	Extinction coefficient: 0.0057 (5)

### Special details

**Table:**

Geometry. All esds (except the esd in the dihedral angle between two l.s. planes) are estimated using the full covariance matrix. The cell esds are taken into account individually in the estimation of esds in distances, angles and torsion angles; correlations between esds in cell parameters are only used when they are defined by crystal symmetry. An approximate (isotropic) treatment of cell esds is used for estimating esds involving l.s. planes.
Refinement. Refinement of F^2^ against ALL reflections. The weighted R-factor wR and goodness of fit S are based on F^2^, conventional R-factors R are based on F, with F set to zero for negative F^2^. The threshold expression of F^2^ > 2sigma(F^2^) is used only for calculating R-factors(gt) etc. and is not relevant to the choice of reflections for refinement. R-factors based on F^2^ are statistically about twice as large as those based on F, and R- factors based on ALL data will be even larger.

### Fractional atomic coordinates and isotropic or equivalent isotropic displacement parameters (Å^2^)

**Table:**

	*x*	*y*	*z*	*U*_iso_*/*U*_eq_	
O1	0.91699 (9)	0.36786 (8)	0.32916 (5)	0.02713 (19)	
H1	0.835 (2)	0.3191 (18)	0.3429 (10)	0.051 (5)*	
O2	0.81808 (10)	0.45251 (9)	0.47136 (5)	0.0329 (2)	
O3	0.85502 (10)	0.68009 (8)	0.44013 (4)	0.0308 (2)	
N1	0.97355 (13)	0.50581 (12)	0.09965 (6)	0.0373 (3)	
N2	0.85183 (11)	0.57435 (10)	0.20247 (5)	0.0263 (2)	
N3	0.81168 (11)	0.67258 (10)	0.14453 (5)	0.0265 (2)	
N4	0.88970 (15)	0.69505 (13)	0.01404 (6)	0.0382 (3)	
H4A	0.8193 (19)	0.7639 (17)	0.0066 (9)	0.039 (4)*	
H4B	0.917 (2)	0.6404 (18)	−0.0258 (10)	0.049 (4)*	
C1	0.88946 (13)	0.62675 (12)	0.08448 (6)	0.0286 (3)	
C2	0.94526 (15)	0.47729 (14)	0.17358 (7)	0.0346 (3)	
H2	0.9802 (18)	0.3965 (17)	0.2035 (9)	0.043 (4)*	
C3	0.78317 (13)	0.58436 (12)	0.27861 (6)	0.0246 (2)	
H3	0.7800 (16)	0.6852 (14)	0.2914 (8)	0.027 (3)*	
C4	0.61677 (13)	0.52484 (12)	0.27637 (6)	0.0266 (2)	
C5	0.55667 (14)	0.43446 (13)	0.21732 (7)	0.0315 (3)	
H5	0.6185 (16)	0.4141 (14)	0.1720 (8)	0.030 (3)*	
C6	0.40816 (16)	0.37325 (15)	0.22196 (8)	0.0409 (3)	
H6	0.372 (2)	0.3122 (18)	0.1811 (10)	0.051 (4)*	
C7	0.31994 (16)	0.40186 (17)	0.28540 (9)	0.0460 (3)	
H7	0.220 (2)	0.3540 (19)	0.2888 (10)	0.056 (5)*	
C8	0.37591 (15)	0.49637 (17)	0.34260 (8)	0.0436 (3)	
H8	0.311 (2)	0.5198 (18)	0.3888 (10)	0.056 (5)*	
C9	0.52284 (14)	0.55881 (15)	0.33796 (7)	0.0349 (3)	
H9	0.5614 (19)	0.6306 (17)	0.3805 (9)	0.045 (4)*	
C10	0.89794 (13)	0.51219 (11)	0.34175 (6)	0.0250 (2)	
H10	1.0024 (15)	0.5580 (13)	0.3377 (7)	0.025 (3)*	
C11	0.85120 (13)	0.54268 (12)	0.42518 (6)	0.0263 (2)	
C12	0.81350 (16)	0.72230 (14)	0.51908 (7)	0.0344 (3)	
H12A	0.6980 (19)	0.7131 (16)	0.5195 (9)	0.041 (4)*	
H12B	0.8654 (17)	0.6543 (16)	0.5579 (9)	0.039 (4)*	
C13	0.8721 (2)	0.86853 (16)	0.53308 (10)	0.0515 (4)	
H13A	0.839 (2)	0.905 (2)	0.5884 (13)	0.077 (6)*	
H13B	0.821 (2)	0.932 (2)	0.4918 (12)	0.076 (6)*	
H13C	1.000 (3)	0.870 (2)	0.5340 (11)	0.071 (6)*	

### Atomic displacement parameters (Å^2^)

**Table:**

	*U*^11^	*U*^22^	*U*^33^	*U*^12^	*U*^13^	*U*^23^
O1	0.0277 (4)	0.0259 (4)	0.0285 (4)	0.0019 (3)	0.0070 (3)	0.0016 (3)
O2	0.0381 (5)	0.0354 (4)	0.0258 (4)	−0.0044 (3)	0.0064 (3)	0.0033 (3)
O3	0.0387 (5)	0.0307 (4)	0.0232 (4)	0.0009 (3)	0.0049 (3)	−0.0016 (3)
N1	0.0413 (6)	0.0472 (6)	0.0247 (5)	0.0175 (5)	0.0113 (4)	0.0041 (4)
N2	0.0274 (5)	0.0307 (5)	0.0214 (4)	0.0049 (4)	0.0073 (4)	0.0028 (4)
N3	0.0308 (5)	0.0279 (5)	0.0217 (4)	0.0019 (4)	0.0077 (4)	0.0030 (4)
N4	0.0515 (7)	0.0397 (6)	0.0250 (5)	0.0125 (5)	0.0141 (5)	0.0052 (4)
C1	0.0296 (6)	0.0335 (6)	0.0235 (5)	0.0025 (4)	0.0072 (4)	0.0005 (4)
C2	0.0376 (7)	0.0414 (7)	0.0257 (6)	0.0159 (5)	0.0087 (5)	0.0035 (5)
C3	0.0279 (5)	0.0272 (5)	0.0196 (5)	0.0034 (4)	0.0079 (4)	0.0009 (4)
C4	0.0249 (5)	0.0307 (6)	0.0244 (5)	0.0052 (4)	0.0039 (4)	0.0050 (4)
C5	0.0298 (6)	0.0357 (6)	0.0289 (6)	0.0053 (5)	0.0013 (5)	0.0023 (5)
C6	0.0333 (7)	0.0462 (8)	0.0419 (7)	−0.0008 (5)	−0.0065 (5)	0.0003 (6)
C7	0.0253 (6)	0.0625 (9)	0.0501 (8)	−0.0028 (6)	0.0026 (6)	0.0103 (7)
C8	0.0279 (6)	0.0658 (9)	0.0382 (7)	0.0027 (6)	0.0099 (5)	0.0052 (6)
C9	0.0286 (6)	0.0480 (7)	0.0290 (6)	0.0047 (5)	0.0074 (5)	0.0007 (5)
C10	0.0246 (5)	0.0270 (5)	0.0237 (5)	0.0001 (4)	0.0042 (4)	0.0005 (4)
C11	0.0251 (5)	0.0298 (6)	0.0237 (5)	−0.0004 (4)	0.0013 (4)	0.0005 (4)
C12	0.0376 (7)	0.0423 (7)	0.0236 (6)	0.0048 (5)	0.0043 (5)	−0.0059 (5)
C13	0.0712 (11)	0.0410 (8)	0.0414 (8)	0.0027 (7)	−0.0013 (7)	−0.0099 (6)

### Geometric parameters (Å, º)

**Table:**

O1—C10	1.3968 (13)	C4—C9	1.3992 (16)
O1—H1	0.881 (18)	C5—C6	1.3939 (18)
O2—C11	1.2079 (14)	C5—H5	0.982 (14)
O3—C11	1.3277 (14)	C6—C7	1.384 (2)
O3—C12	1.4679 (13)	C6—H6	0.938 (17)
N1—C2	1.3242 (15)	C7—C8	1.380 (2)
N1—C1	1.3648 (15)	C7—H7	0.963 (18)
N2—C2	1.3319 (15)	C8—C9	1.3867 (18)
N2—N3	1.3794 (13)	C8—H8	1.017 (18)
N2—C3	1.4609 (13)	C9—H9	1.028 (16)
N3—C1	1.3288 (14)	C10—C11	1.5278 (15)
N4—C1	1.3610 (15)	C10—H10	0.993 (13)
N4—H4A	0.887 (16)	C12—C13	1.486 (2)
N4—H4B	0.898 (18)	C12—H12A	0.983 (16)
C2—H2	0.954 (16)	C12—H12B	1.000 (15)
C3—C4	1.5171 (15)	C13—H13A	1.06 (2)
C3—C10	1.5522 (15)	C13—H13B	1.00 (2)
C3—H3	0.982 (14)	C13—H13C	1.08 (2)
C4—C5	1.3871 (17)		
			
C10—O1—H1	111.7 (11)	C8—C7—C6	119.90 (13)
C11—O3—C12	116.00 (9)	C8—C7—H7	121.3 (10)
C2—N1—C1	102.80 (10)	C6—C7—H7	118.8 (10)
C2—N2—N3	108.99 (9)	C7—C8—C9	120.01 (13)
C2—N2—C3	131.21 (10)	C7—C8—H8	120.6 (10)
N3—N2—C3	119.65 (8)	C9—C8—H8	119.4 (10)
C1—N3—N2	102.53 (9)	C8—C9—C4	120.54 (12)
C1—N4—H4A	115.5 (10)	C8—C9—H9	118.8 (9)
C1—N4—H4B	114.0 (11)	C4—C9—H9	120.6 (9)
H4A—N4—H4B	121.9 (14)	O1—C10—C11	111.63 (9)
N3—C1—N4	123.58 (11)	O1—C10—C3	113.57 (9)
N3—C1—N1	114.34 (10)	C11—C10—C3	111.41 (9)
N4—C1—N1	122.06 (10)	O1—C10—H10	107.7 (7)
N1—C2—N2	111.32 (11)	C11—C10—H10	106.0 (7)
N1—C2—H2	126.7 (9)	C3—C10—H10	106.0 (7)
N2—C2—H2	121.9 (9)	O2—C11—O3	125.05 (10)
N2—C3—C4	112.65 (9)	O2—C11—C10	123.90 (10)
N2—C3—C10	108.22 (8)	O3—C11—C10	111.04 (9)
C4—C3—C10	112.74 (9)	O3—C12—C13	107.49 (11)
N2—C3—H3	106.2 (8)	O3—C12—H12A	106.8 (9)
C4—C3—H3	109.1 (8)	C13—C12—H12A	113.7 (9)
C10—C3—H3	107.6 (8)	O3—C12—H12B	107.6 (9)
C5—C4—C9	118.98 (11)	C13—C12—H12B	111.8 (9)
C5—C4—C3	122.57 (10)	H12A—C12—H12B	109.1 (12)
C9—C4—C3	118.41 (10)	C12—C13—H13A	110.1 (12)
C4—C5—C6	120.04 (12)	C12—C13—H13B	109.0 (12)
C4—C5—H5	120.0 (8)	H13A—C13—H13B	107.4 (17)
C6—C5—H5	120.0 (8)	C12—C13—H13C	109.9 (11)
C7—C6—C5	120.36 (13)	H13A—C13—H13C	108.1 (15)
C7—C6—H6	122.1 (10)	H13B—C13—H13C	112.4 (16)
C5—C6—H6	117.6 (10)		
			
C2—N2—N3—C1	−1.38 (12)	C3—C4—C5—C6	174.36 (11)
C3—N2—N3—C1	−177.37 (10)	C4—C5—C6—C7	−0.12 (19)
N2—N3—C1—N4	−177.46 (11)	C5—C6—C7—C8	2.9 (2)
N2—N3—C1—N1	1.03 (13)	C6—C7—C8—C9	−2.3 (2)
C2—N1—C1—N3	−0.29 (15)	C7—C8—C9—C4	−1.2 (2)
C2—N1—C1—N4	178.23 (12)	C5—C4—C9—C8	3.98 (18)
C1—N1—C2—N2	−0.65 (15)	C3—C4—C9—C8	−173.77 (11)
N3—N2—C2—N1	1.33 (15)	N2—C3—C10—O1	−63.92 (11)
C3—N2—C2—N1	176.70 (11)	C4—C3—C10—O1	61.36 (12)
C2—N2—C3—C4	−94.74 (14)	N2—C3—C10—C11	168.99 (9)
N3—N2—C3—C4	80.22 (12)	C4—C3—C10—C11	−65.74 (12)
C2—N2—C3—C10	30.59 (16)	C12—O3—C11—O2	−0.70 (16)
N3—N2—C3—C10	−154.44 (9)	C12—O3—C11—C10	−179.49 (9)
N2—C3—C4—C5	18.19 (15)	O1—C10—C11—O2	−7.54 (15)
C10—C3—C4—C5	−104.64 (12)	C3—C10—C11—O2	120.60 (12)
N2—C3—C4—C9	−164.15 (10)	O1—C10—C11—O3	171.26 (9)
C10—C3—C4—C9	73.02 (13)	C3—C10—C11—O3	−60.59 (12)
C9—C4—C5—C6	−3.29 (17)	C11—O3—C12—C13	162.57 (11)

### Hydrogen-bond geometry (Å, º)

*Cg*1 is the centroid of the triazole ring.

**Table:**

*D*—H···*A*	*D*—H	H···*A*	*D*···*A*	*D*—H···*A*
O1—H1···N3^i^	0.881 (18)	1.887 (18)	2.7417 (12)	162.9 (16)
N4—H4*A*···O2^ii^	0.887 (16)	2.182 (17)	3.0317 (14)	160.2 (13)
N4—H4*B*···N1^iii^	0.898 (18)	2.127 (18)	3.0066 (15)	166.1 (15)
C2—H2···O1	0.954 (16)	2.257 (16)	2.8665 (14)	120.9 (12)
C12—H12*B*···O1^iv^	1.000 (15)	2.569 (15)	3.4225 (15)	143.2 (11)
C5—H5···*Cg*1	0.982 (14)	2.856 (14)	3.4816 (13)	122.3 (10)

Symmetry codes: (i) −*x*+3/2, *y*−1/2, −*z*+1/2; (ii) −*x*+3/2, *y*+1/2, −*z*+1/2; (iii) −*x*+2, −*y*+1, −*z*; (iv) −*x*+2, −*y*+1, −*z*+1.
